# The relationships between physical function, nutrition, cognitive function, depression, and sleep quality for facility-dwelling older adults with dynapenia

**DOI:** 10.1186/s12877-023-03847-9

**Published:** 2023-05-08

**Authors:** Tzu-Hui Lin, Shu-Fang Chang, Min-Tser Liao, Yen-Hung Chen, Hsiao-Chi Tsai

**Affiliations:** 1 Department of Nursing, College of Nursing, National Taipei University of Nursing and Health Sciences, 365 Ming Te Road, Pei-Tou, 112 Taipei, Taiwan, ROC; 2 Department of Hemodialysis Unit, Taoyuan Armed Forces General Hospital, Hsinchu branch, Hsinchu, Taiwan, ROC; 3 Taoyuan Armed Forces General Hospital, Hsinchu Branch, No. 3, Wuling Rd., North Dist, 300 Hsinchu City, Taiwan, ROC; 4 Department of Information Management, National Taipei University of Nursing and Health Sciences, 112 Taipei, Taiwan, ROC; 5 Cardinal Tien Hospital, No.15, Chezi Rd., Xindian Dist, 23155 New Taipei City, Taiwan, ROC

**Keywords:** Dynapenia, Physical function, Nutrition, Cognitive function, Depression, Quality of sleep

## Abstract

**Background:**

The growing population of older adults worldwide is associated with an extended life expectancy and an increasing proportion of older adults with dynapenia. Most research on dynapenia has involved only populations of older adults living in the community; little research has examined the effects of risk factors on sleep quality among older adults with dynapenia residing in assisted living facilities.

**Aim:**

This study examined the relationships among physical function, nutrition, cognitive function, depression, and sleep quality among older adults with dynapenia residing in assisted living facilities.

**Methods:**

In this cross-sectional study, data on physical function, nutrition, cognitive function, depression, and sleep quality was collected from 178 older adults with dynapenia residing in assisted living facilities, who were selected using purposive sampling. Descriptive statistical analysis, independent-sample *t* tests, chi-squared tests, and logistic regression analysis were performed using SPSS 25.0.

**Results:**

The statistical analyses revealed correlations between sleep quality and age (*t* = 2.37, *p* < 0.05), level of education (χ^*2*^ = 3.85, *p* < 0.05), grip strength (*t* = 3.40, *p* < 0.01), activities of daily living (*t* = 4.29, *p* < 0.001), instrumental activities of daily living (*t* = 2.23, *p* < 0.001), calf circumference (*t* = 2.89, *p* < 0.01), Mini Nutritional Assessment scores (*t* = 2.29, *p* < 0.05), Mini Mental State Exam (MMSE) scores (*t* = 4.50, *p* < 0.001), and Geriatric Depression Scale (GDS) scores (*t* = − 4.20, *p* < 0.001). Calf circumference (OR = 0.8, 95% CI = 0.650.97, *p* < 0.05), GDS score (OR = 1.42, 95% CI = 1.05–1.92, *p* < 0.05), and MMSE score (OR = 0.85, 95% CI = 0.73–0.97, *p* < 0.05) were related to sleep quality among the sample population.

**Conclusion:**

Physical function, nutrition, cognitive function, and depression affect the sleep quality of older adults with dynapenia residing in assisted living facilities. Facility nurses must regularly assess these aspects of their patients to ensure that facility-dwelling older adults can maintain their physical function and improve their health to improve the quality of their sleep.

## Introduction

The global population of individuals over the age of 60 years has increased to over 1 billion. Consequently, geriatric syndromes have become a major health concern [1]. According to statistics compiled by Taiwan’s Ministry of the Interior from 2018 to 2021, in Taiwan, the population over the age of 65 years increased from 3.43 million people (14.6% of the total population) in 2018 to 3.83 million people (16.3% of the total population) by March 2021. Taiwan has been officially considered an aged society since 2018, and, with the population over the age of 65 years expected to increase to 4.7 million people (20.1% of the total population) by 2025, is set to become a super-aged society [2].

Aging results in the deterioration of overall memory and physical strength and can lead to the development of dynapenia. Kobayashi et al. [3] defined dynapenia as a decline in muscular strength or muscle function despite normal muscle mass that affects physical function and mobility, leading to an increased risk of falls and other negative health outcomes among older adults. Benjumea et al. argued that individuals with dynapenia are at a high risk of disability and mortality because decreased muscular strength and mobility may lead to falls [4]. Noh and Park surveyed 2,652 older adults in South Korean communities and discovered that 25.1% of them had dynapenia, which they defined as handgrip strength (HGS) of < 26 kg in men and < 18 kg in women [5]. Kobayashi et al. studied age-related problems among older adults in Japan and discovered that although life expectancy is increasing, individuals’ physical function continues to decrease with age. Defining dynapenia as HGS of < 26 kg in men and < 18 kg in women and a walking speed of < 0.8 m/sec while retaining normal muscle mass (≥ 7.0 kg/m^2^ in men and ≥ 5.7 kg/m^2^ in women), they discovered that the prevalence of dynapenia was 10% [3]. According to statistics compiled by Taiwan’s Ministry of Health and Welfare on the ten leading causes of death in 2018, among individuals aged 65 years or older, falls were the leading cause of death (25.7 people in every 100,000 people), followed by accidental injuries. According to the National Health Survey conducted by the Ministry of Health and Welfare in 2017, one of every six people over the age of 65 years had fallen at least once in the past year. In 2018, individuals over the age of 65 years accounted for 38.2% of health insurance and medical expenses. Evidently, aging is highly correlated with demand for medical treatment, given that an increase in life expectancy within an aging population is associated with an increased demand for medical care. Consequently, determining the key factors affecting the quality of life of older adults is urgent [6].

Pur et al. [7] mentioned that older adults with self-reported physical functioning restrictions have 41% higher odds of having a poor sleep quality (OR = 1.41, 95% CI = 1.22-59, p < 0.05). Moreover, participants having cognitive function problems have 28% higher odds (OR = 1.28, 95% CI = 2.01–3.01, p < 0.05) of having a sleep problem. In addition, nutrition factors play an important role in sleep quality. Sanlier and Sabuncular [8] pointed out that nutrition can profoundly affect the hormones and inflammation status which directly or indirectly contribute to poor sleep quality. Therefore, evaluating the relationship between nutrition and sleep quality was important. Additionally, Wang et al. [9] mentioned that the proportion of poor sleep quality of the elderly in long-term care institutions was significantly higher than that of the elderly in the community (38.2%; χ^2^ = 18.202, p < 0.001), and the higher the degree of depression of the elderly, the worse the sleep quality (β = 0.58, p <. 01). According to the above literature, the physical function, nutrition, cognitive function, and depression of the elderly are related to sleep quality. However, there are still few studies focused on facility-dwelling older adults with dynapenia. Therefore, it is necessary to conduct further analysis.

In addition, researchers have identified correlations between dynapenia and physical weakness and diminished mobility caused by malnutrition, cognitive impairment, and risk of falling. Noh and Park studied the potential connections between dynapenia and mental health and discovered that muscular strength indicators were correlated with mental health risks among older adult men and that low HGS was associated with increased stress among older adult women, indicating that muscular strength may be a crucial clinical indicator of mental health in older adults [5]. A survey commissioned by the Taiwan Society of Sleep Management and conducted by the Chang Gung Hospital Sleep Clinic on trends in the prevalence of common sleep disorders in Taiwan revealed that the prevalence of chronic insomnia in Taiwan is 11.3% and is higher (39.4%) among older adults, with 16.7% and 22.7% of people aged 50–59 and 60–69 years, respectively, experiencing chronic insomnia [10–12]. At present, the relevant literature on sleep quality in the older people with dynapenia is still very rare. Most of the studies were focussed on community dwelling people. Rubio-Arias et al. [13] discovered that dynapenia is associated with age and that sleep quality declines with age; furthermore, the prevalence of dynapenia was significantly higher among older adults who slept less than 6 to 8 h in community dwelling people. Nakakubo et al. [14] collected data on the sleep durations, handgrip strength (HGS), and walking speeds of 3,918 community-dwelling older adults in Japan; among the participants in their study, 10.3%, 73.4%, and 16.3% slept ≤ 6 h, 6.1–8.9 h, and ≥ 9 h, respectively. Their logistic regression analysis revealed that sleep duration and slow gait (OR = 1.55, 95% CI = 1.17–2.06, p = 0.002) were correlated with decreased HGS (OR = 1.34, 95% CI = 1.00–1.78, p = 0.047); in other words, the poorer the quality of an individual’s sleep is, the slower their gait and the weaker their HGS will be.Tseng et al. [15] conducted a study involving 33 facility-dwelling and 55 community-dwelling older adults and discovered significant between-group differences in seven sleep quality domains, with the average sleep quality of the facility-dwelling older adults being poorer than that of the community-dwelling older adults (Mann–Whitney U = 5.09, p < 0.001). In addition, marital status and sleep quality were significantly correlated among the facility-dwelling older adults (Kruskal–Wallis H = 2.224, p < 0.05), and the participants who took sedatives also had poorer sleep quality (Mann–Whitney U = 3.121, p < 0.05). Therefore, facility-dwelling older adults are encouraged to engage in daytime activities and regular exercise and discouraged from lying in bed when not going to sleep.

In summary, these studies indicate that dynapenia may be correlated with sleep quality among community-dwelling older adults. However, few studies have explored dynapenia among older adults residing in care facilities. Therefore, this study conducted a correlational study on sleep quality among older adults with dynapenia residing in care facilities, and the results of the study may serve as a reference for medical staff in developing interventions to improve the sleep quality of facility-dwelling older adults with dynapenia.

## Aim

The aim of this study was to explore the relationships among demographic characteristics, physical functions, nutritional status, cognitive functions, and depression with sleep quality for facility-dwelling older adults with dynapenia.

## Methods

### Research design

This cross-sectional study involved a dynapenia assessment and a survey on physical function, nutritional status, cognitive function, depression, and sleep quality performed with the consent of the older adult participants.

### Participants and setting

In this study, the definition of dynapenia employed Kobayashi et al.— that is, normal muscle mass (≥ 7.0 kg/m^2^ in men and ≥ 5.7 kg/m^2^ in women) and decreased muscular strength (HGS of < 26 kg in men and < 18 kg women) or decreased muscle function (gait of < 0.8 m/s in a 6-meter )—was employed.

Study variables were being considered in this analysis included basic attributes, physical function, nutrition, cognitive function, and depression, and sleep quality. And according to the calculation method of Kobayashi effect size [3]. G*Power 3.1.9.2 indicated that the required sample size for a power of 0.8, medium effect size (0.2), and α level of 0.05 was 178 individuals. Purposive sampling was used to recruit older adults with dynapenia residing in a care facility in northern Taiwan. The inclusion criteria were (1) residence in an assisted living facility, an age of at least 65 years, and dynapenia; (2) the ability to understand and execute simple commands; (3) the ability to communicate clearly and express needs and wishes; and (4) completion of the consent form. The exclusion criteria were (1) inability to communicate in Mandarin or Taiwanese or (2) severe visual or hearing impairment, either of which would prevent the participant from completing the interview/study, and (3) refusal to sign the informed consent form.

### Research tools

#### Dynapenia screening

##### Body composition

Bioelectrical impedance analysis (BIA) was performed using a Danilsmc ioi353 Body Fat Analyzer, which can be used to measure skeletal muscle mass and fat mass in the arms, legs, and torso and is suitable for both community- and facility-dwelling older adults. BIA can clearly identify dynapenia in as little as 10 min and is an inexpensive, fast, and convenient tool that does not require the use of radiation. In a previous study, researchers comparing the effects of psychological and exercise interventions on 57 patients were able to accurately measure changes in body weight, body fat percentage, and skeletal muscle mass using BIA [16]. In another study, researchers used BIA to measure the visceral fat, body fat, and muscle mass of 95 athletes and discovered that BIA exhibited robust internal consistency, with a Cronbach’s α of 0.91 [17].

##### Handgrip strength (HGS)

HGS is the total force produced by the lateral forearm muscles and the medial hand muscles. An individual can measure their HGS (in kilograms) by gripping a dynanometer with one hand under specific conditions. In this study, HGS was measured using a Charder MG4800 handgrip dynanometer, according to which the mean HGS is 30–37 kg for men and 20–25 kg for women. In one study, researchers used the MG4800 dynamometer to accurately measure HGS when examining the association between body mass and sarcopenia among 74 participants with diabetes [18]. In another study, researchers used the MG4800 dynanometer to test the HGS of 406 healthy participants and determined that the test–retest reliability of the dynanometer was 0.98 and the validation results for the MG4800 and Jamar dynanometers were strongly correlated (*r* = 0.954, *p* < 0.05) and strongly consistent (bias – 12.0 N, limit of agreement = − 58.5 to 85.5 N according to Bland–Altman plot) [19].

#### Demographic characteristics

The demographic variables considered in this study were each participant’s sex, age, marital status, education level, religion, history of falls, and medical history.

#### Physical function

##### Biochemical indices

The laboratory data collected included the participants’ *ante cibum* glucose (AC), total cholesterol (TC), total triglyceride (TG), high-density lipoprotein (HDL), low-density lipoprotein (LDL), and creatinine levels.

##### Activities of daily living (ADLs)

The Barthel Index (BI) has been recommended to assess the ADLs of older people [20]. In this study, each patient’s ability to perform ADLs was measured according to seven self-care metrics (feeding, personal hygiene, toilet use, bathing, dressing, bladder control, and bowel control) and three mobility metrics (transferring, walking on level surfaces, and climbing stairs). Each patient received a score of 5 to 15 points for each metric. The highest possible total score was 100 points, which represented functional independence; scores of 91–99, 61–90, 21–60, and 0–20 represented mild, moderate, severe, and total dependence, respectively. The Barthel index [20] was used to assess the ability of patients with neuromuscular or musculoskeletal conditions to perform basic activities in daily life; the internal consistency reliability (Cronbach’s α) was 0.89 [21]. Lin et al. performed an ADL assessment of 67 patients at rehabilitation centers; their overall ADL scores had robust Intra-class correlation coefficient = 0.95; among the individual assessment items, four (personal hygiene, toilet use, bathing, and walking on flat surfaces) exhibited moderate intra-class correlation coefficient = 0.50–0.70, and the remaining six (feeding, transferring, climbing stairs, dressing, bowel control, and bladder control) exhibited robust intra-class correlation coefficient = 0.80–0.95 [22]. Li et al. conducted a retrospective study involving patients with spinal cord injuries and determined that ADL scores can be used to accurately measure correlations between ADLs and time; respondents’ ADL scores tend to decrease over time. The Cronbach’s α of the ADL scale used in that study was 0.93, indicating high reliability [23].

##### Instrumental activities of daily living (IADLs)

The IADL Scale is used to assess more complicated activities in daily life that allow an individual to live independently, such as shopping for groceries and necessities, managing money, taking transportation, cleaning and maintaining their house, managing their health, and engaging in social activities. Current and common older adult quality of life indices have total scores ranging from 0 to 8 and assess a respondent’s ability to shop, engage in activities outside the home, prepare meals, complete chores, do the laundry, use the telephone, take medication, and manage finances. A respondent is considered disabled if they require assistance with three or more of the aforementioned activities. The original IADL Scale was developed by Lawton and Brody to assess the ability of older adults to manage their daily lives and had a validity of 0.61 and a test–retest reliability (Cronbach’s α) of 0.85 [21]. Siriwardhana et al. evaluated the IADL scores of 702 community-dwelling individuals over 60 years old in Sri Lanka and were able to clearly distinguish the community-dwelling older adults’ abilities to engage in ADLs. They determined that the IADL Scale was highly reliable (Cronbach’s α = 0.91) [24].

#### Nutritional status

The participants’ nutritional status was evaluated using the Mini Nutritional Assessment (MNA), which was developed by Guigoz et al. [25] and is considered a comprehensive and easy tool for assessing the nutritional status of older adults. The MNA is highly reliable and can be used by general caregivers and professionals. Furthermore, the MNA is not prone to causing misunderstandings or introducing bias during the data collection process and is a noninvasive, convenient, and rapid nutritional assessment tool, requiring only 10 min to complete. The MNA contains 18 items, and total scores range from 10 to 30 points; scores of ≥ 24, 17–23.5, and < 17 points indicate healthy nutrition, a risk of malnutrition, and malnutrition, respectively [24]. Vellas et al. [26] investigated the reliability and validity of the MNA and determined that the MNA had a sensitivity and specificity of 96% and 98%, respectively, in detecting malnutrition and is therefore appropriate for older populations, community-dwelling older adults, facility-dwelling older adults, and hospitalized patients. Krishnamoorthy et al. used the MNA to assess 278 older adults and concluded that nutrition affects immunity and physical function and that poor nutritional status may lead to increased morbidity and mortality; in the same study, they found the MNA to have satisfactory reliability (Cronbach’s α = 0.71) and recommended it as a tool for assessing the nutritional status of older adults [27].

#### Cognitive function

The cognitive function of the participants was assessed using the 30-item Mini-Mental State Examination (MMSE), which was developed by Folstein et al. for the MMSE evaluates orientation, information registration, attention and calculation, short-term memory, and language; higher scores indicate better cognitive function. In a previous study, the Chinese version of the MMSE was administered to 441 healthy adults and was determined to be highly reliable, with a Cronbach’s α of 0.89 [28]. Amatneeks and Hamdan [29] used the MMSE to survey 163 patients with end-stage renal disease; setting the cutoff for cognitive impairment at ≤ 21 points provided the highest sensitivity (77.46%) and specificity (72.83%) with high reliability (Cronbach’s α = 0.74).

#### Depression

The 15-item Geriatric Depression Scale-Short Form (GDS-SF) was used to evaluate the participants’ depressive symptoms. Respondents answered each item with a *yes* or *no* according to their mood in the past week; higher scores indicate more severe depressive symptoms. The original GDS was developed by Sheikh et al. and used by Greenberg to assess depressive symptoms among older adults; in Greenberg’s study, the GDS had a sensitivity of 0.72 and specificity of 0.57 when used to diagnose depression as well as high reliability (Cronbach’s α = 0.89) [30]. Shimada et al. used the GDS to survey 1,792 older adults with a score of ≥ 6 points indicating depression; of the participants in their study, 13.8% had depression. In that study, the GDS was determined to be highly reliable (Cronbach’s α = 0.86) [31].

#### Sleep quality

The Pittsburgh Sleep Quality Index (PSQI) was developed by Buysse, Reynolds, Monk, Berman, and Kupfer; respondents self-report the quality and quantity of their sleep within the past month in response to 9 items, with item 5 consisting of 10 sub-items. The seven components of the index are subjective sleep quality, sleep latency, sleep duration, habitual sleep efficiency, sleep disturbances, use of sleeping medication, and daytime dysfunction. The total score of subjective sleep quality ranged from 0 to 3 points, where 0 is “very good”, 1 is “good”, 2 is “bad”, and 3 is “very bad”. The higher scores reflect greater sleep impairment. Sleep latency refers to the frequency of respondents being unable to fall asleep within 30 min. The total score of sleep latency ranged from 0 to 3 points, where 0 is “never happened”, 1 is " once time a week”, 2 is " two times a week”, and 3 is “more than or equal to three times a week”. The higher total scores indicate a longer time to falling asleep.

The total score of sleep duration ranged from 0 to 3 points. Zero point means that the total sleep time is more than 7 h. The higher scores reflect the shorter sleep time. The score of habitual sleep efficiency ranged from 0 to 100%. The higher percentage means good sleep. The total sleep disturbance score ranged from 0 to 27. The higher score means the more serious of sleep disturbance. Using of sleeping medication score ranged from 0 to 3. Zero means no sleep medication. The higher score means more frequent in using sleeping pills. Daytime dysfunction score ranged from 0 to 6, where high scores indicates greater daytime dysfunction. With total PSQI scores from 0 to 21, a score of > 5 indicates poor sleep quality, and a score of ≤ 5 indicates good sleep quality; higher scores indicate poorer sleep quality. The two contrasting groups had significantly different global and component scores. Compare to clinic diagnosis and laboratory measurement, a global PSQI score greater than 5 yielded a diagnostic sensitivity of 89.6% and specificity of 86.5% (kappa = 0.75, p < 0.001) in distinguishing good and poor sleepers [32]. Sun et al. surveyed 1,726 70- to 87-year-old community-dwelling older adults and concluded that the PSQI can be used to diagnose sleep disorders in older adults, with an internal consistency reliability (α) of 0.83 and a test–retest reliability of 0.77–0.85; they also determined that sleep quality is associated with age [33].

### Ethical considerations

This study was reviewed and approved by the Institutional Review Board of Taipei City Hospital (IRB number TCHIRB-11,101,014-E). Out of respect for the rights of the participants, the researchers traveled to the facility to introduce themselves to the potential participants and to explain the study—including its objective, inclusion process, contents, and time requirements—before soliciting informed consent from the potential participants. Those willing to participate in the study were required to sign a consent form before participating. All participants gave their written informed consent. If cognitive tests disclosed significant cognitive impairment, written informed consent for participation was provided by a family proxy.

### Data collection

The researchers were responsible for gathering data from the participants. The data collection process was conducted in the facility activity center. The researchers assisted the participants in completing the BIA, grip strength tests, and gait tests and surveyed the physical function, nutritional status, cognitive function, depressive symptoms, and sleep quality of the participants. Regarding to demographics, with the consent of the subjects, institutional nurses assist in providing medical records and collecting research data, and institutional nurses take care of the subjects and fill in the nursing records every day, which can more truly provide the accuracy of the reported data. After the researchers verified that the questionnaires were filled out completely, each participant was given a gift as a token of appreciation for their participation.

### Statistical analysis

Descriptive and inferential statistical analysis, statistical methods include frequency distribution, percentage, mean, standard deviation, minimum value, maximum value, independent-sample *t* tests, chi-squared tests, and logistic regression analysis were performed using SPSS 25.0. In the logical regression analysis, forward selection based on likelihood ratio was used to select the following variables that were significantly correlated with sleep quality and these variables was then analyzed.

In the logical regression analysis, forward selection based on likelihood ratio was used to select the following variables that were significantly correlated with sleep quality: age; education level; HGS; calf circumference; and ADL, IADL, MNA, GDS, and MMSE scores.

## Results

### Demographic characteristics, physical function, nutritional status, cognitive function, depressive symptoms, and sleep quality of facility-dwelling older adults with dynapenia

A total of 222 older people were invited to participate in this study. Nine participants declined and thirteen withdrew. Twenty-two older adults were excluded because they didn’t meet the selection criteria. The study sample comprised 178 facility-dwelling older adults with dynapenia (Table 1), of whom 140 (79%) were men. Because the collection agency of this study is the Home of the Veterans, the main residents are retired soldiers, and the majority of soldiers are male. The participants’ ages ranged between 65 and 102, and the mean age was 81.6. Of the participants, 68 (38.2%), were in the 85- to 94-year age ranges. Most of the participants were married (n = 99, 55.6%). The most common education level was senior high school (n = 53, 29.8%). Most of the participants reported no falls in the past year (n = 149, 83.7%). Regarding the participants’ medical history, 151 had a history of chronic diseases (84.8%), the most common of which was hypertension (n = 116, 65.2%). The participants’ ADL scores ranged from 35 to 100 scores; most (60.1%) of the participants scored 100 (fully independent). The participants’ IADL scores ranged from 0 to 8; of the participants, 135 (75.8%) had no disabilities. The participants’ BMIs ranged from 16.5 to 34.9 kg/m^2^, with a mean of 24.3 kg/m^2^; The participants’ MNA scores ranged from 20 to 30 scores, of the participants, 174 (97.8%) had normal nutritional status. The participants’ MMSE scores ranged from 3 to 30 scores, of the participants, 142 (79.8%) scored 24–30 (indicating normal cognitive function). The participants’ GDS scores ranged from 0 to 15 scores, of the participants, 157 (88.2%) scored 0–5 (in the normal range). The participants’ PSQI scores ranged from 0 to 20 scores, of the participants, 119 (66.9%) scored > 5 (indicating poor sleep quality).

**Table 1:** Descriptive statistics of older adults with dynapenia: demographic characteristics, physical functions, nutritional statuses, cognitive functions, depressive symptoms, and sleep quality (*N* = 178)

Type	Count	Percentage (%)	Range	Mean	SD
Sex					
Male	140	79.0			
Female	38	21.0			
Age group (years)			65–102	81.6	10.2
65–74	62	34.8			
75–84	30	16.9			
85–94	68	38.2			
≥ 95	18	10.1			
Marital status					
Never married	26	14.6			
Married	99	55.6			
Divorced	22	12.4			
Widowed	31	17.4			
Religion					
None	96	53.9			
Buddhism	51	28.7			
Taoism	9	5.1			
Protestant Christianity	15	8.4			
Catholicism	2	1.1			
Folk beliefs	3	1.7			
Other	2	1.1			
Education level					
No education	18	10.1			
Elementary school	44	24.7			
Junior high school	26	14.6			
Senior High school	53	29.8			
Junior college	16	9.0			
College/university	19	10.7			
Graduate school or above	2	1.1			
Fall(s) in the past year					
None	149	83.7			
1	15	8.4			
2	5	2.8			
3	2	1.1			
≥ 4	7	3.9			
History of chronic illnesses					
Yes	151	84.8			
Comorbidities			0–8	1.7	0.9
0	27	15.2			
1	63	35.4			
2	45	25.3			
3	32	18.0			
4	8	4.5			
5	2	1.1			
8	1	0.6			
Cancer					
Yes	15	8.4			
Diabetes					
Yes	47	26.4			
Hyperlipidemia					
Yes	8	4.5			
Gout					
Yes	2	1.1			
Hypertension					
Yes	116	65.2			
Heart disease					
Yes	28	15.7			
Cerebrovascular disease					
Yes	21	11.8			
Dementia					
Yes	8	4.5			
Presenile organic psychosis					
Yes	1	0.6			
Emphysema or chronic obstructive pulmonary disease					
Yes	7	3.9			
Asthma					
Yes	3	1.7			
Peptic ulcers					
Yes	9	5.1			
Bowel dysfunction					
Yes	7	3.9			
Liver disease					
Yes	1	0.6			
Kidney disease					
Yes	8	4.5			
Arthritis					
Yes	14	7.9			
Osteoporosis					
Yes	4	2.2			
Biochemical markers					
*Ante cibum* blood sugar (AC)			71–189	104.8	24.5
Total cholesterol (TC)			107–265	165.2	32.0
Total triglycerides (TG)			39–341	115.9	61.4
High-density lipoproteins (HDL)			24.4-109.1	52.3	15.7
Low-density lipoproteins (LDL)			14.4-187.5	89.7	29.9
Creatine			0.48–10.39	1.2	1.0
ADL			35–100	90.9	15.1
Fully independent, 100 points	107	60.1			
Mild disability, 91–99 points	20	11.2			
Moderate disability, 60–90 points	39	21.9			
Severe disability, 30–60 points	12	6.7			
IADL			0–8	6.9	2.1
No disability	135	75.8			
Disability	43	24.2			
Height			140–177	161.1	7.8
Weight			36.1–97.2	63.0	10.0
Calf circumference			25–38	31.6	1.7
BMI			16.5–34.9	24.3	3.4
Underweight, < 18.5	3	1.7	16.5–18.4	17.5	1.0
Normal, 18.5–24	93	52.3	18.7–24	21.9	1.3
Overweight, 24–27	51	28.7	24.2–27	25.6	0.8
Obese, > 27	31	17.4	27.1–34.9	29.7	2.2
MNA			20–30	27.0	1.8
Normal	174	97.8			
Risk of malnutrition	4	2.3			
MMSE			3–30	23.9	5.1
Normal cognitive function, 24–30 points	142	79.8			
mild cognitive dysfunction, 18–23 points	22	12.4			
Severe cognitive dysfunction, 0–17 points	14	7.8			
GDS			0–15	2.5	2.7
Normal range, 0–5 points	157	88.2			
Mild depression, 6–9 points	14	7.9			
Referral to psychiatry, ≥ 10 points	7	3.9			
Sleep quality			0–20	7.9	4.0
Poor, ≥ 5 points	119	66.9			
Good, ≤ 5 points	59	33.2			
Muscle mass			5.9–12.5	8.2	1.0
Grip strength			5.4–42.3	21.9	8.4
Gait (6-meter walk test)			5.0-15.21	9.0	1.9

Abbreviation description: ADL=Activities of Daily Living Scale; IADL=Instrumental Activities of Daily Living Scale; BMI=Body Mass Index;MNA=Mini Nutritional Assessment; MMSE=Mini-Mental State Examination; GDS=Geriatric Depression Scale; PSQI= Pittsburgh Sleep Quality Inventory

### Sleep quality component scores

The participants’ sleep quality component scores are presented in Table 2. The participants’ mean scores on the subjective sleep quality, sleep latency, sleep duration, habitual sleep efficiency, sleep disturbance, sleeping medication, and daytime dysfunction components were 1.0 (SD = 0.8), 1.4 (SD = 1.1), 1.5 (SD = 1.0), 1.5 (SD = 1.3), 1.1 (SD = 0.6), 1.0 (SD = 1.4), and 0.4 (SD = 0.8), respectively. The high component scores for sleep duration and habitual sleep efficiency indicated that these two areas of sleep quality were the greatest problems for the participants; the participants appeared to have experienced minimal daytime dysfunction.

**Table 2:** Sleep quality component scores (*N* = 178)

	Scores	Count	Percentage	Range	Mean	SD
Subjective sleep quality				0–3	1.0	0.8
Very good	0	50	28.1			
Good	1	90	50.6			
Bad	2	25	14.0			
Very bad	3	13	7.3			
Sleep latency				0–3	1.4	1.1
0 marks	0	48	27.0			
1–2 points	1	61	34.3			
3–4 points	2	25	14.0			
5–6 points	3	44	24.7			
Sleep duration				0–3	1.5	1.0
＞7 h	0	41	23.0			
＞6 h ≤ 7 h	1	28	15.7			
＞5 h ≤ 6 h	2	90	50.6			
＜5 h	3	19	10.7			
Habitual sleep efficiency				0–3	1.5	1.3
≥85%	0	64	36.0			
84%-75 ％	1	29	16.3			
74%-65 ％	2	19	10.7			
＜65%	3	66	37.0			
Sleep disturbance				0–3	1.1	0.6
0 marks	0	15	8.4			
1–9 points	1	124	69.7			
10–18 points	2	38	21.3			
19–27 points	3	1	0.6			
Use of sleeping medication				0–3	1.0	1.4
Never happened	0	113	63.5			
＜once a week	1	4	2.2			
1–2 times a week	2	4	2.2			
≥3 times a week	3	57	32.1			
Daytime dysfunction				0–3	0.4	0.8
0 marks	0	140	78.7			
1–2 points	1	20	11.2			
3–4 points	2	11	6.2			
5–6 points	3	7	3.9			

### Differences in demographic characteristics, physical function, nutritional status, cognitive function, depressive symptoms, and sleep quality among facility-dwelling older adults with dynapenia

The cut-off to group in age, scholarity, and marital status was based on the statistical data of the Taiwan Ministry of the Interior [33], the young and strong elderly are 65–84 years old and the elderly are ≥ 85 years old. In addition, since Taiwan’s national compulsory education is 9 years, it is distinguished by Junior high school or lower and High school or higher. Meanwhile, marital status was simply classified as married or unmarried to explore the differences in sleep quality among older adults with different demographic characteristics.

The chi-square statistical analysis of age group and sleep quality reached a significant difference (χ2 = 5.71, p < 0.05) and the proportion of older people aged 65–84 with good sleep quality was higher than that of those over 85 years old. In addition, chi-square statistical analysis of education level and sleep quality showed a significant difference (χ2 = 3.85, p < 0.05) and the proportion of older people with high school or higher level with good sleep quality was higher than that of junior high school or lower. Meanwhile, chi-square statistical analysis of ADL level and sleep quality has a significant difference (χ2 = 14.86, p < 0.01) and the proportion of fully independent older people with good sleep quality was higher than that of non-fully independent people. Additionally, the chi-square statistical analysis of IADL level and sleep quality reached a significant difference (χ2 = 9.42, p < 0.01) and the proportion of incapacitated people with good sleep quality is higher than that of disability people. Chi-square statistical analysis of MMSE level and sleep quality showed a significant difference (χ2 = 10.05, p < 0.01), and the proportion of normal cognitive older people with good sleep quality was higher than that of those without normal cognitive function (Table 3).

**Table 3:** Differences in sleep quality among older adults with different demographic characteristics, physical function, nutritional statuses, cognitive function, and depressive symptoms (*N* = 178; nominal variables)

Variable	Quality of sleep					
	Good		Poor		χ^*2*^	*p*
	*N*	(%)	*N*	(%)		
Sex					1.017	.313
Male	49	(35)	91	(65)		
Female	10	(26)	28	(74)		
Age group (years)					5.719*	.017
65–84	38	(41)	54	(59)		
≥ 85	21	(33)	65	(67)		
Marital status					0.491	.484
Never Married/Divorced/widowed	24	(30)	55	(70)		
Married	35	(36)	64	(65)		
Religion					1.783	.182
No	36	(38)	60	(62)		
Yes	23	(28)	59	(72)		
Education level					3.859*	.049
Junior high school or lower	23	(26)	65	(74)		
High school or higher	36	(40)	54	(60)		
Falls within the past year					0.483	.487
No	51	(34)	98	(66)		
Yes	8	(28)	21	(72)		
Chronic illnesses					0.178	.673
No	8	(30)	19	(70)		
Yes	51	(34)	100	(66)		
ADL					14.862**	.002
Fully independent, 100 points	45	(42)	62	(58)		
Mild disability, 91–99 points	8	(40)	12	(60)		
Moderate disability, 60–90 points	5	(13)	34	(87)		
Severe disability, 30–60 points	1	(8)	11	(92)		
IADL					9.425**	.002
No disability	53	(39)	82	(61)		
Disability	6	(14)	37	(86)		
BMI					3.439	.329
Underweight, < 18.5	1	(25)	2	(75)		
Normal, 18.5–24	26	(28)	67	(72)		
Overweight, 24–27	22	(43)	29	(57)		
Obese, > 27	10	(32)	21	(68)		
MNA					0.123	.726
Normal	58	(33)	116	(67)		
Risk of malnutrition	1	(25)	3	(75)		
MMSE					10.051**	.007
Normal cognitive function, 24–30 points	55	(39)	87	(61)		
Mild cognitive dysfunction, 18–23 points	3	(14)	19	(86)		
Severe cognitive dysfunction, 0–17 points	1	(7)	13	(93)		
GDS^a^					4.789	.092
Normal range, 0–5 points	56	(35)	101	(65)		
Mild depression, 6–9 points	3	(27)	11	(73)		
Referral to psychiatry, ≥ 10 points	0	(0)	7	(100)		

Note: *p < 0.05, **p < 0.01, ***p < 0.001;^a^*p* value is for fisher’s exact test

Abbreviation description: ADL = Activities of Daily Living Scale; IADL = Instrumental Activities of Daily Living Scale; BMI = Body Mass Index; MNA = Mini Nutritional Assessment; MMSE = Mini-Mental State Examination; GDS = Geriatric Depression Scale

As indicated in Table 4, the average age of the participants with poor sleep quality was higher than that of the participants with good sleep quality (*t* = − 2.37, *p* < 0.05). Compared with the participants with good sleep quality, those with poor sleep quality had worse ADL scores (*t* = 4.29, *p* < 0.001), worse IADL scores (*t* = 2.23, *p* < 0.001), lower MMSE scores (*t* = 4.50, *p* < 0.001), higher GDS scores (*t* = − 4.20, *p* < 0.001), lower MNA scores (*t* = 2.29, *p* < 0.05), a smaller average calf circumference (*t* = 2.89, *p* < 0.01), and weaker HGS (*t* = 3.40, *p* < 0.01) (Table 4).

**Table 4:** Differences in sleep quality among older adults with different demographic characteristics, physical functions, nutritional statuses, cognitive functions, and depressive symptoms (*N* = 178; continuous variables)

	Quality of sleep			
Items	Good *N* (*M ± SD*)	Poor *N* (*M ± SD*)	*t*	*p*
Age	59(79 ± 10.24)	119(82.82 ± 10.03)	-2.378*	.018
Biochemical markers				
*Ante cibum* blood sugar (AC)	42(102.14 ± 27.74)	80(106.18 ± 24.23)	-0.830	.408
Total cholesterol (TC)	42(159.14 ± 27.72)	80(168.31 ± 33.79)	-1.511	.133
Total triglycerides (TG)	42(110.95 ± 55.47)	80(118.44 ± 64.40)	-0.639	.524
High-density lipoproteins (HDL)	42(52.11 ± 12.61)	80(52.43 ± 17.11)	-0.109	.914
Low-density lipoproteins (LDL)	42(84.84 ± 26.81)	80(92.31 ± 31.20)	-1.315	.191
Creatine	42(1.35 ± 1.48)	80(1.14 ± 0.62)	1.131	.260
ADL	59(96.44 ± 8.91)	119(88.19 ± 16.69)	4.295***	.000
IADL	59(7.59 ± 1.19)	119(6.49 ± 2.29)	2.235***	.000
MMSE	59(25.88 ± 3.16)	119(22.92 ± 5.58)	4.505***	.000
MNA	59(27.46 ± 1.99)	119(26.8 ± 1.70)	2.297*	.023
GDS	59(1.58 ± 1.61)	119(3.02 ± 2.95)	-4.206***	.000
Comorbidities	59(1.65 ± 1.183)	119(1.75 ± 1.35)	-0.500	.618
Skeletal muscle of limbs	59(21.422 ± 3.63)	119(21.164 ± 3.53)	0.454	.650
Grip strength	59(24.825 ± 8.53)	119(20.427 ± 7.90)	3.406**	.001
Gait speed	41(8.5776 ± 1.76)	57(9.25 ± 1.98)	1.737	.086
Z score of body height	59(-0.06 ± 1.01)	119(0.03 ± 0.99)	-0.56	.574
Z score of body weight	59(0.04 ± 1.04)	119(-0.02 ± 0.98)	0.35	.724
Z score of calf circumference	59(0.36 ± 1.34)	119(-0.18 ± 0.72)	2.89**	.005

*p < 0.05, **p < 0.01, ***p < 0.001

Abbreviation description: ADL = Activities of Daily Living Scale; IADL = Instrumental Activities of Daily Living Scale; BMI = Body Mass Index; MNA = Mini Nutritional Assessment; MMSE = Mini-Mental State Examination; GDS = Geriatric Depression Scale

### Relationship with demographic characteristics, physical function, nutritional status, cognitive function, and depressive symptoms for sleep quality among older adults with dynapenia

As indicated in Table 5, calf circumference was significantly correlated with sleep quality, with ORs of < 1 indicate a large calf circumference and good sleep quality. GDS scores were also significantly correlated with sleep quality, with ORs of > 1 indicating a high GDS score and poor sleep quality; furthermore, increases in GDS scores were associated with 1.42 times the risk of poor sleep quality. MMSE scores were significantly correlated with sleep quality, with ORs of < 1 indicating strong cognitive function and good sleep quality.

**Table 5:** The relationships between physical function, nutrition, cognitive function, depression, and sleep quality for facility-dwelling older adults with dynapenia. (*N* = 178)

Independent variable	B	SE	OR	(95%CI)	Wald	*p*
Calf circumference	-0.228	0.103	0.80	(0.65–0.97)	4.914*	0.027
GDS	0.349	0.155	1.42	(1.05–1.92)	5.053*	0.025
MMSE	-0.173	0.073	0.85	(0.73–0.97)	5.604*	0.018

*p < 0.05, **p < 0.01, ***p < 0.001

Abbreviation description: MMSE = Mini-Mental State Examination; GDS = Geriatric Depression Scale

## Discussion

In summary, calf circumference, GDS scores, and MMSE scores can be used as relationship with sleep quality among older adults with dynapenia. The participants in this study were facility-dwelling older adults over the age of 65 years, most of whom were in the 85–94 age group, with an average age of 81.6 years. Most of the participants were male, married, high school–educated, nonreligious, and not prone to falling. The majority had chronic illnesses, the most common of which was hypertension. These findings are similar to those of the study on facility-dwelling older adults conducted by Tseng et al. [15], in which most of the participants were married and had chronic illnesses.

Regarding physical function, over half of the participants were determined to be fully independent or without disabilities according to their ADL and IADL scores. This finding is consistent with that of the study Maximo et al., in which recurrent fallers with dynapenia were weaker and exhibited greater functional deterioration, although over half were assessed to be fully independent or without disabilities according to the ADL and IADL scales [11]. Furthermore, in the present study, 97.8% of the participants had normal nutrition. This finding is inconsistent with that of the study by Chen et al., in which 43.5% of the participants had malnutrition. This inconsistency may be due to different old age residential settings. [34]. Chen et al. studied the nutritional status of community-dwelling older adults who were discharged from hospital 6 to 9 months ago and who were responsible for their own meals; by contrast, the facility at which the participants in the present study resided has nutritionists on staff to ensure the nutritional balance of the meals offered, and the participants in the present study had better nutritional status than did those in the study by Chen et al. Regarding cognitive function, most of the participants had MMSE scores corresponding to normal cognitive function; this finding was similar to that of the study by Chen et al., in which 63% of the participants had normal MMSE scores[34]. The overwhelming majority of participants in the present study did not have depression according to their GDS scores, which is consistent with the study by Maximo et al., in which 83.1% of the participants did not have depression [11]. This indicates that older adults with dynapenia are not more prone to depression than are older adults without dynapenia. Moreover, the results of this study indicate that over half of older adults with dynapenia have poor sleep quality, echoing the findings of another study involving patients at a hospital outpatient clinic for older adults, in which 63.2% of the participants had poor sleep quality [35]. These findings are also consistent with those of Abeysekera and De Zoysa, who discovered that most facility-dwelling older adults in Sri Lanka have poor sleep quality [36].

This study revealed differences in sleep quality among older adults with dynapenia with different ages; education levels; calf circumferences; ADL, IADL, GDS, MMSE, and MNA scores. Compared with the participants with good sleep quality, the participants with poor sleep quality had a higher average age, lower average ADL and IADL scores, and a higher average GDS score. Noguchi et al. studied the associations among physical function, depression, and quality of sleep among 300 community-dwelling older adults with an average age of 73 years in Japan and discovered significant correlations among age, IADL and GDS scores, and sleep quality [37]. Hsu et al. studied sleep problems, depression, health conditions, and physical functions among 400 older adult patients at a hospital clinic in southern Taiwan and identified significant associations among ADL scores, GDS scores, and sleep quality; in that study, compared with the participants with good sleep quality, those with poor sleep quality were generally less educated and had lower MMSE scores [35]. These findings, alongside the findings of Liu et al., who discovered that level of education and cognitive function were significantly correlated with sleep quality of among 6,828 older adults over the age of 50 years, and those of Song et al., who identified a significant correlation between cognitive function and sleep quality among 144 veterans, were consistent with the findings of the present study [38, 39]. In the present study, the comparison of calf circumference, HGS, and sleep quality revealed that the participants with smaller calf circumferences had poorer sleep quality; this is consistent with the findings of Denison et al., who discovered that declining physical function, gait, and HGS were significantly associated with declining sleep quality among 401 older adults, and those of Li et al., who identified an association between lower HGS and poorer sleep quality in a study involving 1,142 older adults. In addition, in the present study, poorer nutritional status was correlated with poorer quality of sleep, which is consistent with the conclusion of Liu et al. that malnutrition is significantly associated with poor sleep quality among older adults [40–42]. Dynapenia develops gradually with age but should not be regarded as a natural sign of aging. Instead, it must be viewed as a chronic disease and treated accordingly. A review of the relevant literature yielded little evidence of the effects of physical function, nutrition, and cognitive function on sleep quality of older adults with dynapenia residing in assisted living facilities (hereafter “facility-dwelling older adults”); to mitigate this gap, this study invested the relationship with dynapenia among this group. The results may serve as a reference for institutional nurses in providing care to patients with dynapenia, reducing the burden of medical care and improving the quality of sleep of older adults in assisted living facilities.

According to the results of the present study, calf circumference, GDS scores, and MMSE scores are strong related to sleep quality. Previous studies have also reported that calf circumference is associated with sleep quality. Ueshima et al. [43] evaluated the calf circumference index proposed by the Asian Working Group for Sarcopenia in 2019 and discovered that a calf circumference below the cutoff was indicative of insufficient muscle mass was associated with negative health outcomes. Nakakubo et al. observed correlations among decreasing gait speed, declining HGS, and poor sleep quality and deduced that as a result of calf muscle atrophy, older adults with dynapenia may become slower and therefore be discouraged from engaging in activities, which may negatively affect their sleep quality [14]. Liu et al. conducted a study involving 224 community-dwelling older adults in Tianjin, China and discovered that HGS, gait speed, and sleep quality were correlated and that more severe depressive symptoms were associated with poorer sleep quality among the older adults with dynapenia [44]. Noguchi et al. and de Paula Reboucas et al., who conducted a study of 220 community-dwelling older adults in Brazil, also arrived at similar conclusions. Furthermore, in the present study, stronger cognitive function, as indicated by higher MMSE scores, was associated with better sleep quality [37, 45]. These findings are similar to those of Liu et al., who identified a correlation between cognitive function and sleep quality among adults over the age of 50 years in China, and Zhou et al., who conducted a study involving 84 older adults with dementia and 92 healthy older adults and discovered a correlation between cognitive function and sleep quality among the older adults with dementia [44, 46]. Study the role of prevention and rehabilitation strategies in improving sleep quality in these patients, such as adopting multi-channel interventions, such as: using more brains, increasing brain stimulation classes, learning new knowledge; more exercise, walking, aerobics and aerobics; Stay away from depression, take adequate rest, cultivate interests, find someone to talk to, and maintain a happy mood [47].

This study has limitations. First, the participants were recruited through purposive sampling; therefore, the inferences that can be drawn from the results may be limited. Second, the demographics, history of falls, and medical history were based on the participants’ self-report and medical record. Therefore, the accuracy of study was limit. Third, most of the older people in this research institution are retired soldiers who receive national pension care. The institution provided rehabilitation therapists, nutritionists, nurses, social workers, and doctors to provide comprehensive care for these older people. Therefore, most of they had no falls, were fully independent on ADLs, had normal cognition on the MMSE, and nearly all had normal nutrition. These factors were examined in relation to sleep quality, the association might restrict because of insufficient range of scores. Moreover, because the study employed a cross-sectional design, the causal relationships among physical function, nutritional status, cognitive function, depressive symptoms, and sleep quality could not be fully explained. In the future, researchers should conduct longitudinal studies to track the risk factors affecting sleep quality among older adults with dynapenia and determine how to help such individuals improve the quality of their sleep.

## Conclusion

The results of this study indicate that older adults with dynapenia generally have a poor quality of sleep. Furthermore, the sleep quality of older adults with dynapenia varies among individuals with different ages; education levels; HGS; calf circumferences; and ADL, IADL, MNA, MMSE, and GDS scores. In other words, when older adults with dynapenia begin to exhibit declining physical activity or cognitive function or experience depressive symptoms, their quality of sleep will significantly worsen. Furthermore, this study revealed that calf circumference and GDS and MMSE scores can be used to poor sleep quality. Facility nurses should assess older adults with dynapenia on these major risk factors to help them improve their quality of sleep. It is necessary to further investigate the role of prevention and rehabilitation strategies to improve the quality of sleep in these older persons.

Most of the older people in this research institution are retired soldiers who receive national pension care, and the institution has rehabilitation therapists, nutritionists, nurses, social workers and doctors to provide comprehensive care, so most of them did not fall, completely independent ADL, and MMSE. With normal cognition, almost all people have normal nutrition. It is also known that the limitations of this study are the characteristics of the Home of the Veterans, and that the subjects received good medical care.

## Data Availability

Raw data of the current study can be provided upon request. Tzu-Hui Lin could be contacted if someone wants to request the data from this study.
